# Validation of a rapid remote digital test for impaired cognition using clinical dementia rating and mini-mental state examination: An observational research study

**DOI:** 10.3389/fdgth.2022.1029810

**Published:** 2022-12-21

**Authors:** Ali Alim-Marvasti, Narayan Kuleindiren, Kirsten Harvey, Matteo Ciocca, Aaron Lin, Hamzah Selim, Mohammad Mahmud

**Affiliations:** ^1^ Research Division, Mindset Technologies Ltd., London, United Kingdom; ^2^ Queen Square Institute of Neurology, University College London, London, United Kingdom; ^3^ Department of Medical Physics and Biomedical Engineering, University College London, London, United Kingdom; ^4^ Department of Brain Sciences, Imperial College London, London, United Kingdom; ^5^ Medical School, University of Birmingham, Birmingham, United Kingdom

**Keywords:** MCI (mild cognitive impairment), early cognitive decline, smartphone application, stroop, delayed recall, digit symbols modality test, CDR, MMSE-2

## Abstract

**Background:**

The Clinical Dementia Rating (CDR) and Mini-Mental State Examination (MMSE) are useful screening tools for mild cognitive impairment (MCI). However, these tests require qualified in-person supervision and the CDR can take up to 60 min to complete. We developed a digital cognitive screening test (M-CogScore) that can be completed remotely in under 5 min without supervision. We set out to validate M-CogScore in head-to-head comparisons with CDR and MMSE.

**Methods:**

To ascertain the validity of the M-CogScore, we enrolled participants as healthy controls or impaired cognition, matched for age, sex, and education. Participants completed the 30-item paper MMSE Second Edition Standard Version (MMSE-2), paper CDR, and smartphone-based M-CogScore. The digital M-CogScore test is based on time-normalised scores from smartphone-adapted Stroop (M-Stroop), digit-symbols (M-Symbols), and delayed recall tests (M-Memory). We used Spearman's correlation coefficient to determine the convergent validity between M-CogScore and the 30-item MMSE-2, and non-parametric tests to determine its discriminative validity with a CDR label of normal (CDR 0) or impaired cognition (CDR 0.5 or 1). M-CogScore was further compared to MMSE-2 using area under the receiver operating characteristic curves (AUC) with corresponding optimal cut-offs.

**Results:**

72 participants completed all three tests. The M-CogScore correlated with both MMSE-2 (rho = 0.54, *p *< 0.0001) and impaired cognition on CDR (Mann Whitney *U* = 187, *p *< 0.001). M-CogScore achieved an AUC of 0.85 (95% bootstrapped CI [0.80, 0.91]), when differentiating between normal and impaired cognition, compared to an AUC of 0.78 [0.72, 0.84] for MMSE-2 (*p *= 0.21).

**Conclusion:**

Digital screening tests such as M-CogScore are desirable to aid in rapid and remote clinical cognitive evaluations. M-CogScore was significantly correlated with established cognitive tests, including CDR and MMSE-2. M-CogScore can be taken remotely without supervision, is automatically scored, has less of a ceiling effect than the MMSE-2, and takes significantly less time to complete.

## 1. Introduction

Worldwide prevalence of cognitive impairment is rapidly increasing, including that of mild cognitive impairment (MCI). Despite having important implications for patients and families, MCI is underdiagnosed in primary care. According to the Alzheimer association, only 16% of patients who are 65 years or older are screened for cognitive impairment, even though this is listed as an integral and standard component of the annual wellness visit for patients with Medicare insurance (1). Detecting MCI before the onset of dementia allows individuals time to sort their affairs and it also supports the recruitment of individuals with early neurodegenerative disorders into disease-modifying clinical pharmaceutical trials (2).

Accessing specialised memory units for the screening of cognitive impairment has been increasingly difficult, with the COVID-19 pandemic further impacting access, especially amongst the elderly. Screening tests such as the Mini-Mental State Examination Second Edition Standard Version (MMSE-2) can take 15 min or longer to complete (3, 4). The clinical dementia rating scale (CDR) requires the physical presence of a companion that knows the patient well, and can take up to 60 min to complete (5). Both the MMSE and CDR additionally require certified administrators to carry out the testing. Telemedicine and digital technologies have been advocated to improve patients' assessment and support (6). There is therefore an increasing need for remote digital solutions to replace in-person supervised cognitive screening, especially for the detection of early cognitive impairment (7, 8).

The ideal remote digital solution would not require supervision, could be completed remotely, would have good test-retest reliability, take less time to complete, would have sensitivity and specificity comparable to existing tests that screen for MCI, and would have a clear and strong theoretical grounding in established neuropsychological evaluations. Such criteria are difficult to achieve for the following reasons: 1) the results of a digital solution would need to be shown to be correlated with established cognitive screening tests in healthy controls, as well as in age, sex, and education matched individuals with early cognitive impairments (9, 10); 2) unlike the MMSE, a good remote digital screening test would be freely accessible (11); 3) shortening the duration of screening is usually associated with fewer items on the test, lower sensitivity for mild impairments, and a potential collapse of the multi-dimensional testing of different cognitive domains (12). Therefore, detecting MCI, compared to detecting dementia, usually requires longer questionnaires and more time (8).

In this study we focused on detecting early cognitive impairments using a rapid digital solution that could surmount the aforementioned difficulties. The M-CogScore is based on three shortened tests that evaluate different cognitive domains: the Stroop test that measures cognitive flexibility and ability to inhibit interference (13, 14), digit symbols modality test evaluating processing speed, attention and working memory (15, 16), and delayed recall (17). We set out to validate the Mindstep M-CogScore digital solution using the MMSE-2 and CDR in the context of an observational research study. We administered all three tests to participants in this study. A CDR score of 0.5 or 1 was taken as the benchmark suggesting early impaired cognition (18, 19). The new digital M-CogScore showed convergent validity with the established MMSE-2, and discriminative validity using CDR.

## 2. Methods

### 2.1 Overview

We used the Mindstep remote digital cognitive screening tool (M-CogScore), which results in an automated score. The cognitive screening is based on smartphone application-adapted Stroop, digit-symbols, and memory recall tests that accounts for both accuracy and the time taken to complete the tests. The M-CogScore was administered as part of an observational research study, along with the CDR and MMSE-2, to healthy controls and to those with impaired cognition.

The well-established CDR test was administered to all participants and their companions by a certified administrator. The CDR global score (range 0–3) was calculated, where 0 was normal, and anything over 0 was impaired cognition (18). All participants also took the 30-item MMSE-2 and were given a score out of 30 (3).

CDR is an established and widely used rating scale for staging patients with cognitive impairment. First used in 1982 and subsequently revised, CDR stratifies patients in five categories, based on their score in six different cognitive and behavioural domains, with CDR global scores calculated *via* an algorithm and higher scores corresponding to more severe impairment (18, 20). As the definition of MCI was firstly proposed by Petersen and colleagues in 1999 (21), CDR scores of 0.5 and 1 were originally purported to identify patients with “questionable” (CDR 0.5) or “mild dementia” (CDR 1). Some studies have used a CDR global score of 0.5 to indicate the presence of MCI, while others have suggested that patients with MCI and mild Alzheimer's disease may be staged with a CDR score of 0.5 or 1 (22). Therefore, we decided to consider this variability in our study by recruiting patients with CDR global scores of 0 for healthy controls and 0.5 or 1 for cognitive impairment, recognising that our group may not only encompass patients with MCI but may include those with generally impaired cognition.

### 2.2. Observational research study

#### 2.2.1. Recruitment criteria

Participants were recruited from local databases at four clinical research sites in the United Kingdom: Guildford, Birmingham, London, and Plymouth. Inclusion criteria were age ≥ 45 years, having the capacity to provide informed consent, and a CDR global score of 0, 0.5, or 1. Exclusion criteria were the presence of any condition or state that would affect the participant's ability to complete the study: neurological disorders, psychiatric conditions, medication use, and/or substance use.

#### 2.2.2. Participants

50 healthy controls (confirmed with CDR global score of 0) and 55 participants with impaired cognition or MCI (CDR 0.5–1) were recruited, matched for age, sex, and level of education.

All participants attended the testing site accompanied by a companion who knew them well for the CDR examination. Each participant, and their companion, gave written informed consent to participate in the study.

#### 2.2.3. Setting and data collection

All participants attended the study site in person and completed the testing over the course of one day between November 2021 and May 2022. Participants were tested in a private room by a certified administrator who was experienced in performing the CDR and MMSE-2 in participants with impaired cognition. The testing session lasted up to two hours, with breaks permitted as required.

The testing session began with the administration of the CDR to the participant and their companion, to determine inclusion to either the healthy control (global score 0) or impaired cognition groups (global score 0.5 or 1). Given the sensitivity of the subject matter, the companions took part in the CDR assessment in a private room and their answers were not shared with the participant, thus providing an environment where the companions could speak freely. To prevent distraction or assistance, the companions were not permitted to be present during the participant's testing session. Any participant who scored 2 or above was classified as a screening failure and did not complete the rest of the testing procedures.

The enrolled participants were administered the 30-item standard version of the second edition of the MMSE (MMSE-2) and were scored out of 30. They were also asked a series of predefined questions on their demographics, education, lifestyle, and medical history which were recorded onto a paper case report form. The participants age, sex, and education were used to ensure matching between the two groups.

Finally, the participant self-administered the Mindstep app, which includes digital cognitive screening and records details of the participant's medical history, age, education, and lifestyle factors, as previously described (23, 24). Mindstep has been previously validated as an iPhone iOS remote data acquisition application that can replicate known epidemiological findings (23).

The Mindstep app was pre-downloaded onto an iPhone 10 provided at the research site for the participant to use. If a participant was not an experienced iPhone user, they were given time to familiarise themselves with how to use it, practising with another app prior to starting the testing. The Mindstep app is designed to be used without clinical supervision and therefore the participants were not given any assistance during digital testing. They were observed from a distance to ensure that they completed the testing, and the administrator recorded any additional relevant information for the researchers. The administrators were blinded to the results of the M-CogScore.

All participant data were pseudonymised at the trial site after collection, then uploaded and stored in secure Amazon Web Services (AWS) servers.

#### 2.2.4. Ethics

This study involved human participants and was given favourable ethical opinion by West Midlands - Solihull Research Ethics Committee (21/WM/0202). Participants and their companions gave informed consent to participate in the study before taking part. All researchers underwent Good Clinical Practice training and procedures were conducted in accordance with General Data Protection Regulation and the 1964 Declaration of Helsinki and later amendments.

### 2.3. Digital cognitive score (M-cogScore)

The M-CogScore is based on three shortened tests that evaluate different cognitive domains: the *Stroop test* that measures cognitive flexibility and ability to inhibit interference (13, 14), *digit symbols modality test* evaluating processing speed, attention and working memory (15, 16), and *delayed recall*. Below we explain how M-CogScore derives from theoretical grounding in each of these three established neuropsychological evaluations. All three tests were answered using the Apple iPhone 10s, where participants used touch to either pick the correct option from a list (Stroop and Memory) or handwrite the answers on screen (Symbols). Participants self-administered the app with only technical guidance as necessary.

#### 2.3.1. Memory

The delayed free recall of lists of words is frequently used to detect cognitive impairment. Several versions have been used, sometimes with fewer words to make the test quicker and easier to administer (26). The test battery of the Consortium to Establish a Registry for AD (CERAD) comprises a ten-word list and has been shown to be one of the more sensitive tests for detecting MCI. It consists of a learning phase of an immediate-recall trial, an inter-trial interference task, and a delayed-recall trial (2). The diagnosis of cognitive impairment can be based on a combination of the first and second recall trials, or on a cut-off score of the delayed-recall trial (2).

The Mindstep Memory test (M-Memory) involved remembering eight words over two consecutive presentations, preceded by an in-app demonstration. The first presentation flashed each word on the screen for one second. After the first presentation, there was an immediate cued recall trial with four multiple choices available for each item, of which three of the words were distractors. Subsequently, there was an immediate second presentation of the eight words, again each one flashing on the screen for one second. The second presentation of the eight words was followed by the M-Stroop and M-Symbols as interference tests, before returning to recall the items. At the second cued recall trial there were eight multiple choices available for each item, with seven of these used as distractors. Neither the first nor the second cued recall trials gave feedback on the answers.

M-Memory score was calculated using the total number correct out of eight (accuracy). Time was not included in the calculation to decorrelate this score from the time denominators in the other two scores (M-Stroop and M-Symbols).

#### 2.3.2. Stroop

During a standard Stroop task, participants are asked to name the colour of text in which a word is written, instead of reading the text that spells a colour. The text could be either congruent or incongruent with the colour. This test generates the so-called Stroop effect, defined as a delay in reaction time between congruent and incongruent stimuli (14). The Stroop test measures a combination of the ability to inhibit cognitive interference, the capacity for selective attention, processing speed, and cognitive flexibility (13). Stroop scores have been calculated in different ways, such as accuracy within a fixed period of time, or using a combination of accuracy and speed from amongst a set number of items (13). M-CogScore utilised the latter methodology.

Mindstep Stroop (M-Stroop) involved eighteen trials of the coloured texts: “red”, “green”, and “blue”. The user had to identify the colour from a list of three options. The colours and text were congruent in six trials, and incongruent in twelve trials. The M-Stroop score was calculated by dividing the total number correct by the time taken, a measure dependent on both accuracy and speed (27).

#### 2.3.3. Symbols

The Symbol Digit Modalities Test is a quick and reliable neuropsychological test to assess processing speed (15). It consists of a key of symbols matched to unique numbers, in which participants are required to match a list of given symbols to their corresponding numbers within a specific time frame. Usually, the number of correct answers constitutes the raw score (16). In addition to processing speed, the Symbol Digit Modalities Test assesses attention, visual scanning and tracking, and working memory (15).

The Mindstep Symbols (M-Symbols) test involved matching nine symbols to their corresponding number before writing the answers on screen. These were presented in three trials of three symbols. A custom neural network algorithm was used to automatically identify the handwritten number, and this was converted to a digital number and compared to the answer. The M-Symbols test score was calculated using the number of symbols correct, divided by the time taken to complete the test.

#### 2.3.4. M-CogScore

All scores were calculated after user data had been uploaded to AWS servers. The overall M-CogScore was calculated by taking the mean of the z-scores for M-Stroop, M-Symbols, and M-Memory scores, in line with previous neuropsychological batteries that averaged the z-scores of subtests (8). This mean constituted the overall M-CogScore. Participants were not coerced to complete all sections and were able to skip any section if necessary. M-CogScore was however only calculated when scores for all sections were available.

### 2.4. Analysis of incomplete or missing data

The full analysis was performed on all participants for whom data for all three tests (CDR, MMSE-2, M-CogScore) were available. If participants were not included in the full analysis due to incomplete or missing data, this was quantified to check which tests they did not complete, and what the average scores of any completed tests were. Average ages of excluded participants were also compared with included participants.

### 2.5. Statistical analyses

Spearman's correlation coefficient (rho) was used to assess the strength of inter-score correlations, as both MMSE-2 and M-CogScore were not normally distributed (Shapiro-Wilk *p *< 0.05). Non-parametric Mann-Whitney U tests were used for comparisons with bivariate CDR data (healthy controls defined as CDR of 0, vs. mild impaired cognition CDR of 0.5 or 1). The area under the receiver operating characteristic curves (AUC) were computed for distinguishing between normal and impaired cognition, with optimal sensitivity and specificity cut-off thresholds for MMSE-2 and M-CogScore calculated using Youden's J statistic. 10,000 bootstrapped samples with a 0.75 proportion were used to obtain the 95% confidence intervals of the AUCs. The non-parametric DeLong method was used to compare differences between AUCs (28).

Baseline categorical demographics between the two groups were compared using chi-squared or Fisher's exact tests (if any value or expected cell value was 5 or less). Continuous normally distributed data were compared using t-tests, while non-Gaussian continuous data were compared using the Mann-Whitney U (MWU) non-parametric test. Shapiro-Wilk tests were used to test for normality with an alpha threshold of <0.05 suggesting the variable was not normally distributed.

The difference in median scores between the two groups (healthy controls and impaired cognition) were normalised to the overall range of scores separately for both MMSE-2 and M-CogScore:

$$\;\mathrm{proportional}\;\mathrm{difference}\;\mathrm{in}\;\mathrm{median}\;\mathrm{scores}\;=\frac{\mathrm{Difference}\;\mathrm{in}\;\mathrm{median}\;\mathrm{scores}}{\mathrm{Maximum}\;\mathrm{score}-\mathrm{Minimum}\;\mathrm{score}}$$

Statistical significance threshold was set at 0.05. Statistical analyses were performed using python v 3.9, pandas v 1.4.1, scipy v 1.6.2, matplotlib v 3.5.1, and seaborn v 0.11.2 (29).

### 2.6. Data availability

Anonymised analysis-ready data and the statistical analyses are available upon reasonable request by contacting the corresponding author on: alijesus.alim-marvasti@nhs.net. The digital cognitive test is freely available to download from: https://apps.apple.com/gb/app/mindsteps/id1530813748.

## 3. Results

### 3.1. Participants and demographics

**Figure 1:**
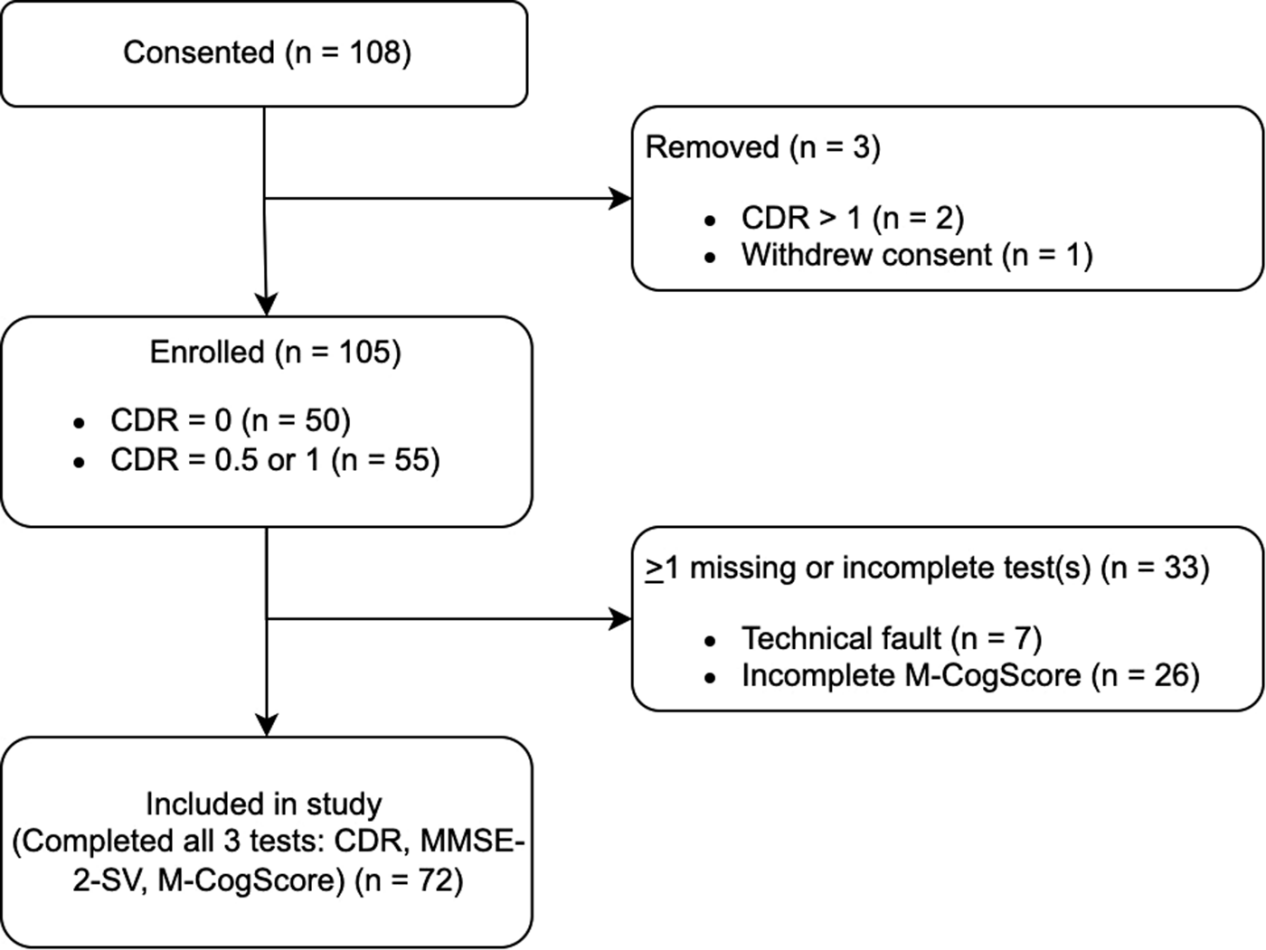
Participant flow diagram. There were 26 incomplete data for M-CogScore. Additionally, 7 participants’ *M-CogScore* data failed to upload to Amazon Web Services Cloud due to issues with internet connection.

Initially, 108 participants consented, of which one later revoked consent. Two participants scored in the more severe dementia categories on the CDR global scores (CDR > 1), their data was therefore omitted as per exclusion criteria. Of the remaining 105, seven participants' M-CogScore data failed to upload to the AWS cloud due to a failed internet connection, and a further 26 skipped parts of the M-CogScore resulting in the automated scoring mechanism labelling them as incomplete without giving a total score (see sensitivity analyses and limitations). Therefore, a total of 72 participants were included (Figure 1).

72 participants completed all tests (MMSE-2, CDR, M-CogScore, Figure 1), of which 34 had impaired cognition (1 CDR = 1; 33 CDR = 0.5). Mean age in the impaired cognition group was 69.5 ± 6.6 years, and in the healthy controls was 69.1 ± 5.5 years.

As we matched for sex, age, and education during recruitment, none of these were found to be significantly different between healthy controls and individuals with impaired cognition (Table 1).

**Table 1:** Demographics of all participants and summary MMSE-2 and M-CogScore results by CDR score.

	Healthy Controls (*n* = 38) (CDR = 0)	Impaired Cognition (*n* = 34) (CDR 0.5 or 1)
Male, Female	17, 21	15, 19
Age		
Mean ± std	69.1 ± 5.5 years	69.5 ± 6.6 years
Median [IQR]	69 [66, 72]	69.5 [65.25, 74.75]
(min, max)	(53, 85)	(57, 81)
Education		
No formal education	2	4
GCSE	9	4
A-levels	6	12
University	21	14
MMSE-2 (/30)		
Mean ± std	28.6 ± 1.2	26.1 ± 2.8
Median [IQR]	29 [28, 30]	27 [24, 28]
(min, max)	(26, 30)	(20, 30)
M-CogScore		
Mean ± std	0.47 ± 0.47	−0.52 ± 0.77
Median [IQR]	0.55 [0.19, 0.82]	−0.38 [−1.13, −0.01]
(min, max)	(−0.48, 1.31)	(−1.91, 0.97)

There was no significant difference between healthy controls and individuals with impaired cognition in their sex (Fisher’s exact test *p* = 0.81), age (*t*-test *p* = 0.73), or highest level of education (MWU = 583, *p* = 0.22).

Std: standard deviation; IQR, interquartile range; CDR, clinical dementia rating scale global score; GCSE, general certificate of secondary education (high school diploma in United Kingdom); A-levels, advanced level (pre-university).

Both MMSE-2 and M-CogScore scores were lower in the impaired cognition group (*p *< 0.0001, Table 1), which can also be seen in the plots in Supplementary Figure S1.

### 3.2. Digital and standard cognitive screening tests

**Figure 2:**
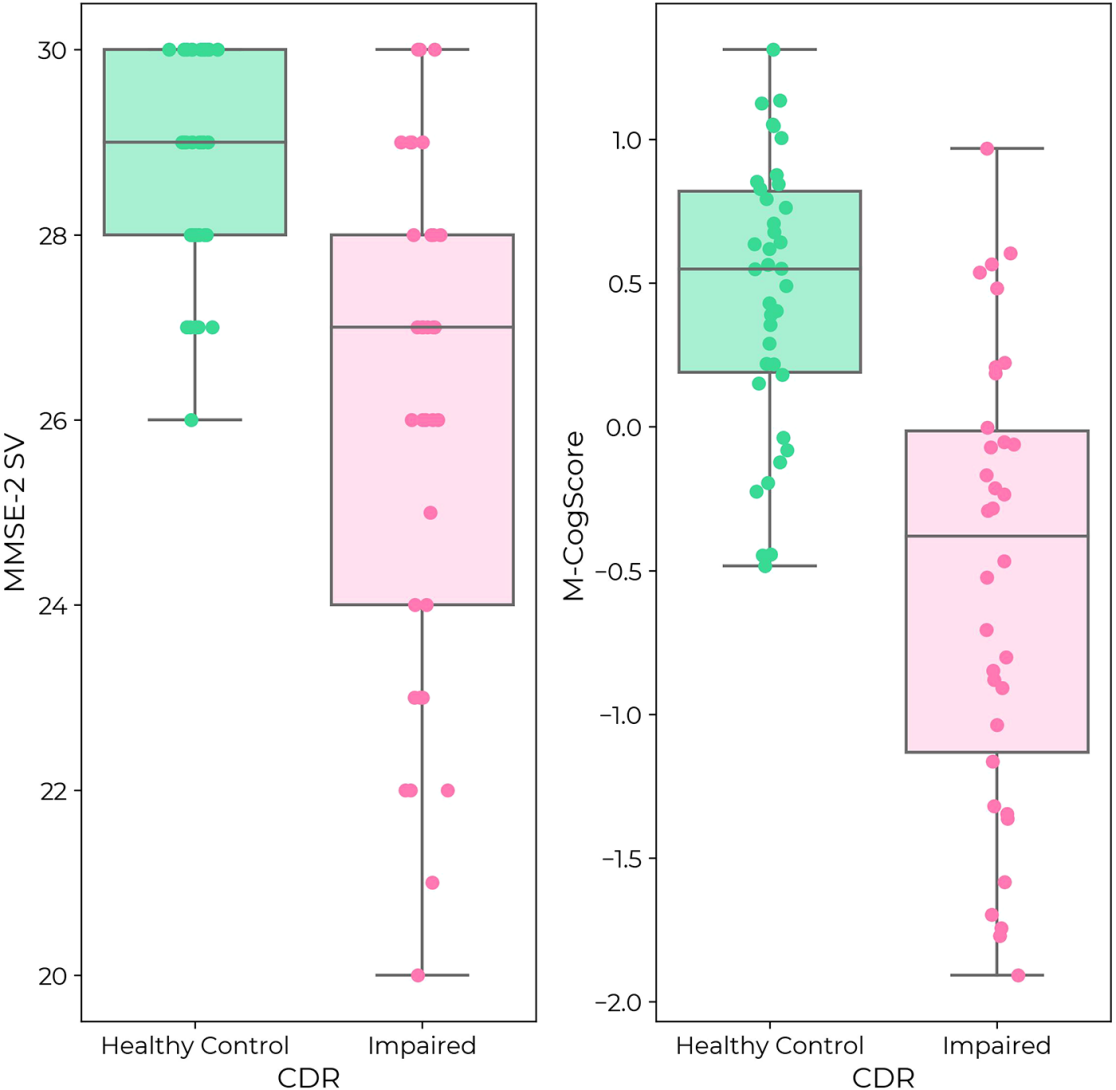
**(Left)** MMSE-2 in healthy controls (green) and individuals with impaired cognition (pink) according to CDR label. **(Right)** M-CogScore in healthy controls (green) and impaired cognition (pink). Both MMSE-2 and M-CogScore were significantly lower in individuals with impaired cognition (*p* < 0.0001). While there was a ceiling effect for MMSE-2 in healthy controls, there was no ceiling effect for M-CogScore.

MMSE-2 scores and CDR global scores were correlated (*p *< 0.0001, Figure 2). Median MMSE-2 scores in participants with impaired cognition (CDR 0.5 or 1: Median 27/30, IQR [24, 28]) were 2 points lower than in healthy controls, which was 20% of the total range of scores (CDR = 0: Median 29/30, IQR [28, 30]) (MWU = 287, *p *< 0.0001). There was a clear ceiling effect of the MMSE-2 in healthy controls, but not in those with impaired cognition (Figure 2).

M-CogScores and CDR global scores were also correlated (*p *< 0.0001, Figure 2). M-CogScores in participants with impaired cognition (CDR 0.5 or 1, Median −0.38, IQR [−1.13, −0.01]) were on average 0.93 points lower than in healthy controls, which was 47.7% of the total range of scores (CDR = 0, Median 0.55, IQR [0.19, 0.82]) (MWU = 189 *p *< 0.0001).

Unlike the MMSE-2, there was no ceiling effect of the M-CogScore in healthy controls (Figure 2).

### 3.3. Digital cognitive test: M-cogScore and MMSE-2

**Figure 3:**
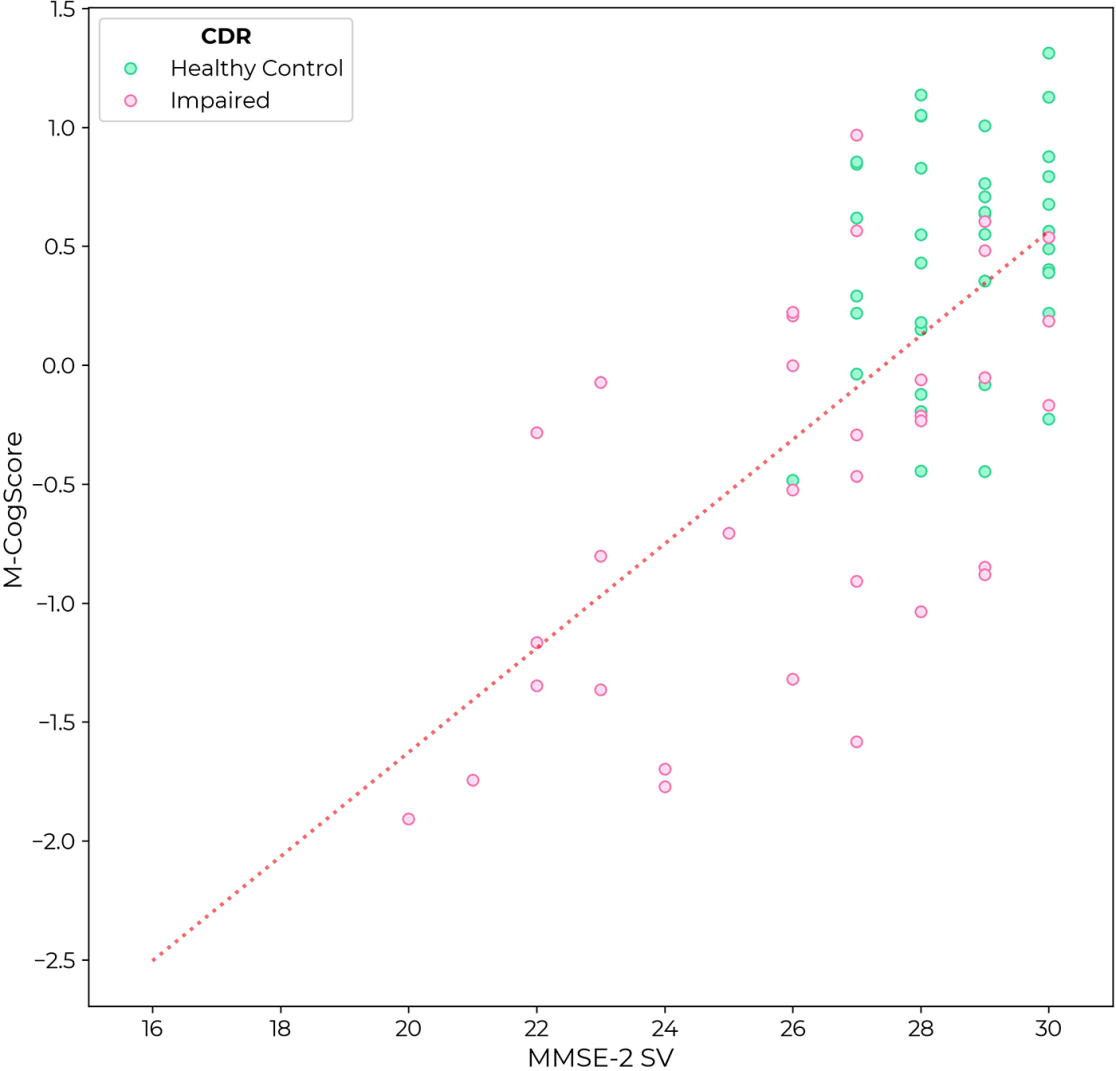
M-CogScore vs. MMSE-2 across all participants, labelled by CDR as controls (CDR = 0, green) or impaired cognition (CDR 0.5 or 1, pink).

M-CogScore was correlated with MMSE-2 (rho = 0.54, *p *< 0.0001). Again, unlike in the MMSE, there was no ceiling effect in M-CogScores in healthy controls (Figure 3).

### 3.4. Receiver operating characteristic curves

**Figure 4:**
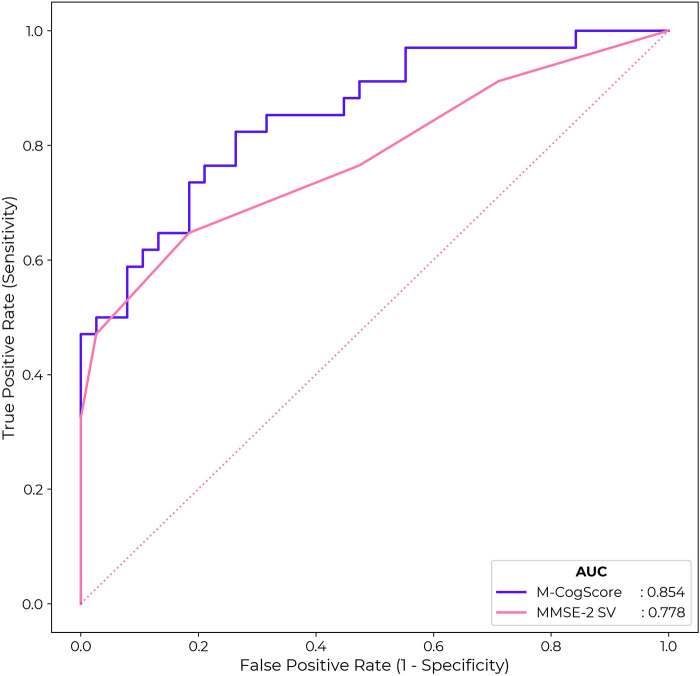
ROC for M-CogScore and MMSE-2 to detect impaired cognition based on CDR scores. M-CogScore AUC of 0.85 was non-significantly greater than that of MMSE-2 0.78 (DeLong *p* = 0.21). The optimal threshold for MMSE-2 was 27 and for M-CogScore 0.21.

Receiver operating characteristic curves (ROC) for M-CogScore and MMSE-2 are shown in Figure 4. The M-CogScore's AUC was 0.85 (95% bootstrapped CI [0.80, 0.91]), which was non-inferior to that of the MMSE-2 AUC of 0.78 [0.72, 0.84] (DeLong *p *= 0.21).

Cut-off thresholds for the optimal trade-off in sensitivity and specificity, to detect impaired cognition based on CDR global scores, were < 27 for MMSE-2 and < 0.21 for M-CogScore. This cut-off of 27 for the MMSE-2 gave a sensitivity of 0.65 and specificity of 0.82. The threshold of 0.21 for the M-CogScore gave a sensitivity of 0.82 with a specificity of 0.74.

### 3.5. Analysis of incomplete or missing data

Of the 33 participants for whom data was not available for all three tests (CDR, MMSE-2, and M-CogScore), 26 had an incomplete M-CogScore. Of these 26, 9 were found to have incomplete M-Stroop scores, 5 had incomplete M-Symbols scores, and 12 had incomplete M-Memory scores, because these sections of the M-CogScore had been skipped by participants. 7 participants' M-CogScore data failed to upload to the AWS cloud due to internet connection issues, and this data was unrestorable (Figure 1).

Of the total 33 with missing data, 12 were healthy controls (mean age 71.0 years ± 3.3) and 21 had impaired cognition (mean age 71.6 years ± 7.3; 15 CDR = 0.5 and 6 CDR = 1). Although mean ages for both groups were slightly older than their included counterparts (Table 1), neither reached statistical significance (t-test, *p *= 0.12 and 0.76, respectively). The 12 healthy controls scored a mean of 27.6 ± 1.5 on the MMSE-2, while the 21 with impaired cognition scored a mean of 23.7 ± 4.3. Both groups on average scored less on the MMSE-2 than their included counterparts for whom all three test scores were available, as seen in Table 1 (MWU, *p *= 0.02 for both). Overall, the proportion of participants with impaired cognition from amongst the 33 with missing data was not significantly higher than that of included participants (64% vs. 47%, Chi-squared, *p *= 0.18).

In the incomplete M-CogScore data subgroup with 26 participants, 7 were healthy controls (CDR = 0) while 19 had impaired cognition (6 with CDR = 1, 13 with CDR = 0.5). Both the healthy controls and impaired cognition groups, on average, scored less on the MMSE-2 than their included counterparts for whom all three test scores were available (MWU, *p *= 0.02 and 0.004, respectively). Healthy controls with incomplete data had a mean age of 71 years ± 3.3 with a range of 66–74 years. The incomplete impaired group's mean age was 71.6 years ± 7.3 with a range of 53–85 years. Although individuals with incomplete data were slightly older than participants included in the main analysis (Table 1), this did not reach significance for either the healthy controls or impaired cognition groups (*p *= 0.38 and 0.29, respectively).

The proportion of participants with impaired cognition in the incomplete M-CogScore data subgroup (19/26, 73%) was more than in the main analysis (34/72, 47%; Chi-squared = 4.2, *p *= 0.04).

## 4. Discussion

### 4.1. Overview

The digital M-CogScore showed convergent validity with the established cognitive test MMSE-2 (rho = 0.54, *p *< 0.0001) and discriminative validity using CDR (*p *< 0.0001). M-CogScore was at least as good as MMSE-2 in distinguishing between normal and impaired cognition (M-CogScore AUC = 0.85 vs. MMSE-2 = 0.78).

While the CDR takes up to 60 min (5, 19, 30), and MMSE-2 takes 10–15 min to complete (3), the M-CogScore can be completed in less than 5 min (23). Additionally, although the CDR is used extensively in research trials and epidemiological studies to identify cognitively impaired individuals, it requires the presence of both a trained test-supervisor to administer and score, as well as the presence of a companion (31), which are not required for the M-CogScore. The CDR is partly multiple-choice answers and partly open-ended answers, at times requiring complex clinical judgement to score each section (18, 19). The MMSE-2 also requires a trained administrator but no patient companion, whereas the M-CogScore is taken independently and automatically scored.

This study showed that the short, remote, and digital cognitive M-CogScore test was comparable with both the MMSE-2, which is an updated version of the original and widely administered MMSE cognitive screening test (3), and the CDR, which is a structured cognitive questionnaire without objective testing. These results are particularly noteworthy because short tests are usually considered to be inadequate to detect MCI (12).

Additionally, the finding that the MMSE-2 and CDR scores were correlated in this cohort, replicates existing relationships shown in previous studies, complete with a MMSE-2 cut-off threshold (27/30) to detect impaired cognition that is comparable to established normative data (8, 11, 32). These results indicate that the relationship between MMSE-2 and CDR was preserved in our sample, suggesting that the sample of participants in this study were representative and comparable to populations from previous studies.

However, there was a disproportionate number of incomplete M-CogScore tests. Because this incomplete data pertained to individuals who had impaired cognition on CDR and lower average MMSE-2 scores, difficulty with completing the digital test was associated with cognitive impairment in t cohort. Therefore, M-CogScore may be applicable as an initial screening tool for individuals with normal or mild cognitive impairment only.

### 4.2. Comparison of M-CogScore and established cognitive screening tests

The moderate correlation between M-CogScore and the MMSE-2 found in this study is clinically significant because MMSE-2 is more sensitive to milder cognitive impairments than its original version (3). In this study M-CogScore still had a similar, if not larger, AUC for the detection of impaired cognition (0.85 vs. 0.78, *p *> 0.05). The performance of M-CogScore's AUC of 85% also compares favourably to the original MMSE's performance from the literature. The AUC for the original MMSE to detect MCI in the literature ranges from 67.6% (based on CDR global scores in 144 individuals) (33), or 74% (compared to clinical history and a standard battery of neuropsychological testing in 293 individuals) (8) to at most 82% obtained by combining 21 studies (Table 2) (34). Furthermore, the MMSE-2 used in this study, has a high equivalency with the ubiquitous original 30-item MMSE, especially for the detection of impaired cognition, with no changes to the normal range of scores, and good test-retest reliability as measured by an intraclass correlation coefficient of 0.90 (3).

**Table 2:** Comparisons between cognitive tests in detecting MCI.

Paper Cognitive Tests						
Paper Cognitive Tests	Time to Complete Test	Severity of Cognitive Impairment and Definition	Number of Study Participants	Performance Metric	Value	Reference
MMSE-2	10–15 mins	MCI (CDR = 0.5 or 1)	72	AUC	78%	(This study)
MMSE	10–15 mins	MCI (CDR > 0)	144	AUC	68%	Li, Ng (33)
		MCI (clinical history and a standard battery of neuropsychological testing)	293	AUC	74%	Van Patten, Britton (8)
				Meta-analysis	21 studies	AUC	82%	Tsoi, Chan (34)
MOCA	10–15 mins	Combination of MCI and mild AD	200 (84 healthy, 68 MCI, 48 mild AD)	AUC	82%	Modarres, Khazaie (35)
ACE-III	30 mins	Combination of MCI and mild AD	200 (84 healthy, 68 MCI, 48 mild AD)	AUC	84%	Modarres, Khazaie (35)
Digital Tests
Digital Tests	Time to Complete Test	Severity of Cognitive Impairment and Definition	Number of Study Participants	Performance Metric	Value	Reference
M-CogScore	5 mins	MCI (CDR > 0.5 or 1)	72	AUC	85%	(This study)
Integrated Cognitive Assessment (ICA)	5 mins	Combination of MCI and mild AD	200 (84 healthy, 68 MCI, 48 mild AD)	AUC	91%	Modarres, Khazaie (35)

ACE-III, addenbrooke’s cognitive examination III; MOCA, montreal cognitive assessment; AD, alzheimer’s disease.

M-CogScore's AUC performance of 85% also compares favourably to other cognitive impairment screening tests. For example, the Montreal Cognitive Assessment (MoCA) is a freely available 30-item questionnaire, and the Addenbrooke's Cognitive Examination III (ACE-III) is a 100-item longer questionnaire. Both have been shown to detect MCI in 200 participants (84 healthy controls) with an AUC of 82% and 84% respectively (35). Therefore, the performance of the rapid, remote, and digital M-CogScore screening test is comparable to that in the literature for both the MoCA and ACE (Table 2).

As a cognitive screening test, a high sensitivity (82% for M-CogScore), without significant compromise in specificity (74%), is desirable to ensure early detection of cognitive decline and early recruitment to interventional studies (7). For comparison, a pooled sensitivity of 62% has been reported for the original MMSE (34), congruent with this study's sensitivity of 65% for the MMSE-2, which is lower than the M-CogScore sensitivity of 82%. The original MMSE's pooled sensitivity is quoted as 62% and specificity as 87%, while the alternative MoCA has a pooled sensitivity of 89% and specificity of 75% for the detection of MCI (subgroup analysis of 9 studies) (34). Therefore the sensitivity and specificity of M-CogScore is similar to that of the MoCA from the literature.

Some studies have identified MMSE scores lower than 28/30 as the optimal threshold for detecting MCI (8), while others have recommended a cut-off of 26 (with 21–26 representing MCI) (11, 32), One study on 863 individuals comparing MMSE with CDR global scores and a 50% validation set, identified MMSE scores in the range of 26 to 29 as corresponding to a CDR score of 0.5 but with low agreement (Cohen's kappa = 0.28), compared to higher agreement in more severe cognitive deficits (MMSE of 21 to 25 corresponding to a CDR score of 1, with kappa = 0.62) (36). In line with these previous studies, we found the optimum threshold for MMSE-2 to be 27. This comparable result suggests that not only is the MMSE-2 equivalent to the original MMSE, but also indirectly validates the participants used in this study as a broadly representative sample of the general population for which the tests are intended.

This study used the CDR as the benchmark. CDR is a structured, clinician or technician-rated interview that collects information from both the patient and uniquely also the caregiver or companion in order to stage the severity of cognitive impairment across six domains: memory, orientation, judgement, problem-solving, community affairs, home and hobbies, and personal care (31). The CDR is a well-established screen with fair inter-rater reliability and good test-retest reliability (31, 37). The usefulness of the CDR as a benchmark score is attested to by the fact that increasing CDR scores are correlated with changes in neurodegenerative biomarkers including lower amyloid-beta-42 and higher phosphorylated tau levels in cerebrospinal fluid (38), and with imaging changes including temporal lobe atrophy (39). As per previous studies, this study used CDR global scores (CDR = 0) to define healthy controls (18, 19), and any CDR score more than zero as the threshold benchmark for impaired cognition, which could signify milder impairment than the use of a CDR cut-off of greater than or equal to 1 (37). This threshold has previously been shown to have excellent pooled sensitivity of 93% and specificity of 97% for the detection of MCI (15 studies) (40). By including a CDR score of 0.5 in the category of impaired cognition, we tested the performance and sensitivity of M-CogScore to less severe cognitive impairment. The majority of our participants had a CDR of 0.5.

Short cognitive tests in general have been considered inadequate to detect MCI and the usual modifications commonly involve extending test batteries which invariably take longer to complete (12). Examples of this include the extended version of the MMSE-2 or the modified MMSE (8). A modified, shortened version of the CDR, with greater inter-rater reliability, has been proposed so that it is possible to complete in 10 min, but like the original CDR this too does not directly test cognition, requires both an administrator and test companion, and has not been widely adopted (19). The Integrated Cognitive Assessment (ICA) is also a short digital cognitive test with good convergence validity and AUC of 91% (Table 2) (35). The ICA, however, uses deep learning and a linear classifier to detect normal from mild AD in addition to MCI. The inclusion of mild AD might be expected to improve the sensitivity of tests as it would be easier to distinguish from healthy controls (12), while the use of high-dimensional machine learning is more likely to overfit datasets. Conversely, M-CogScore uses transparent and established tests based on Stroop, Symbols, and Memory, and this study excluded participants with more severe cognitive deficits, but nevertheless still achieved an AUC of 85%. There have also been two recent reviews on the topic of digital cognitive tests for MCI, which summarise findings from other studies (41, 42).

In summary, the M-CogScore is a shorter digital screening test with equivalent performance to the MMSE, MoCA, and ACE, and intended for the detection of early impaired cognition through a remotely administered and automatically scored programme.

### 4.3. M-CogScore: A rapid, remote and automatically scored digital cognitive screening test

Mindstep is a remote digital software that collects information on common risk factors for cognitive impairment and the M-CogScore is administered through this software (23, 24). Mindstep and M-CogScore are intended for unsupervised remote use. However, participants in this study were recruited based on inclusion and exclusion criteria and attended a study centre for completion of both paper-based tests and the Mindstep questionnaires as part of a larger study. Nevertheless, the standalone Mindstep application has demonstrated peer-reviewed validity (23) by replicating previously known epidemiological data for individuals with anxiety and depression (24), and concussion (25), thereby validating the use of the application for remote data collection.

M-CogScore is calculated as the mean of the z-score of three modified tests based on the Stroop, Digit Symbols Modality Test, and delayed recall. Although other cognitive screening tests evaluate similar cognitive domains, speed is not explicitly utilised in the scoring of CDR or MMSE and has a unique role in M-CogScore. Speed of completion directly influences two out of three of the constituent M-CogScore test scores (M-Stroop and M-Symbols). Speed and accuracy can be a proxy for cognitive function (27), and can be an early marker of MCI and change in daily function (43). Digital solutions make accurate measurements of speed easier and more reliable, which may be an important factor in the overall good performance of M-CogScore, even when compared to the established clinical screening tools such as the MMSE-2.

### 4.4. Limitations

Most of the incomplete data came from the M-CogScore application. While incomplete answers or skipping subsections were given a score of zero by test administrators for paper CDR and MMSE tests, the automated scoring method of M-CogScore did not ascribe a score, and therefore marked the data as incomplete. Therefore, there were more incomplete data points from the application, which may have been due to technical difficulties, perhaps related to the M-CogScore being available only on the iPhone which may be unfamiliar to some users, or even more general difficulty using technology that may come with more advanced cognitive deficits. In line with this, we found that those who failed to complete the digital M-CogScore, compared to those who completed it, were more likely to have impaired cognition on CDR screening. Additionally, we found that the MMSE-2 scores of individuals with incomplete M-CogScores were lower. Thus, M-CogScore, may not be suitable for more advanced cognitive impairment and dementia, consistent with its design as a screening tool for early cognitive impairment. Alternatively, future studies using M-CogScore could explore the role that skipping parts of tests can have on the overall score and prediction of cognitive impairment, so as to avoid data loss. This limitation could also be incorporated to M-CogScore's design to detect impaired cognition in primary care, by flagging individuals who were unable to complete the test for formal clinical screening.

The M-CogScore was constructed using the mean of the z-scores of its three constituents, one of which was not normally distributed (Supplementary Figure S2), therefore care must be taken in the interpretation of the absolute value of the M-CogScore. Nevertheless, the M-CogScore displays appropriate normal distributions when split by healthy control individuals and those with impaired cognition (Supplementary Figure S1).

We used CDR as the benchmark for distinguishing between normal and impaired cognition, but CDR does not objectively test cognition and its subjective scores have moderate inter-rater reliability (e.g., Cohen's weighted kappa of 0.56) (19). However, to reduce the impact of CDR's inter-rater variability, we dichotomised scores into 0 or 0.5 and 1 to signify impaired cognition, and additionally correlated scores with the MMSE.

Although participants only had technical supervision at the test centre when completing the M-CogScore, they were in a controlled environment, while it is expected that the app would be used unsupervised at home, which may affect ecological validity. An additional limitation is that M-CogScore is currently aimed for English speakers with access to the Apple iOS.

## 5. Conclusions

M-CogScore is correlated with MMSE-2 (rho = 0.54, *p *< 0.0001) and detects impaired cognition at least as well as the MMSE-2 (AUC 0.85 vs. MMSE 0.78). A threshold of 0.21 in M-CogScore was approximately equivalent to an MMSE-2 score of 27, giving a sensitivity of 0.82 (vs. MMSE-2 0.65) and a specificity of 0.74 (vs. MMSE-2 0.82). While the MMSE-2 had a ceiling effect, M-CogScore did not.

Unlike other artificial-intelligence based solutions, M-CogScore is transpicuous in its formulation as the mean of the z-scores of three modified tests based on the Stroop, Digit Symbols Modality Test, and delayed recall. M-CogScore is applicable as an initial screening tool for individuals with normal or mild cognitive impairment, with difficulty completing the test associated with cognitive impairment in our cohort.

These results are particularly noteworthy because M-CogScore is independently and remotely administered, automatically scored, and can be undertaken in under 5 min on a smartphone to screen for impaired cognition. Reliable and easily understood remote digital screening is important for the early detection of cognitive impairment and recruitment to disease-modifying neurodegenerative studies. Digital technologies have the potential to expedite patients' assessment at scale (6). M-CogScore shows promise as a remote digital solution to replace in-person supervised cognitive screening at scale, especially for the detection of early cognitive impairment (7, 8).

## Data Availability

The raw data supporting the conclusions of this article will be made available by the authors, without undue reservation.
