# Supplementary material for: Validation of a rapid remote digital test for impaired cognition using clinical dementia rating and mini-mental state examination: An observational research study
Source: Front Digit Health. 2022 Dec 21;4:1029810. doi: 10.3389/fdgth.2022.1029810 (PMC9811948; doi:10.3389/fdgth.2022.1029810)
Supplement: Supplementary file 1 [file Datasheet1.docx]

Supplementary Materials

# Results

## Supplementary Figure 1: MMSE-2 and M-CogScore Distributions

**A**


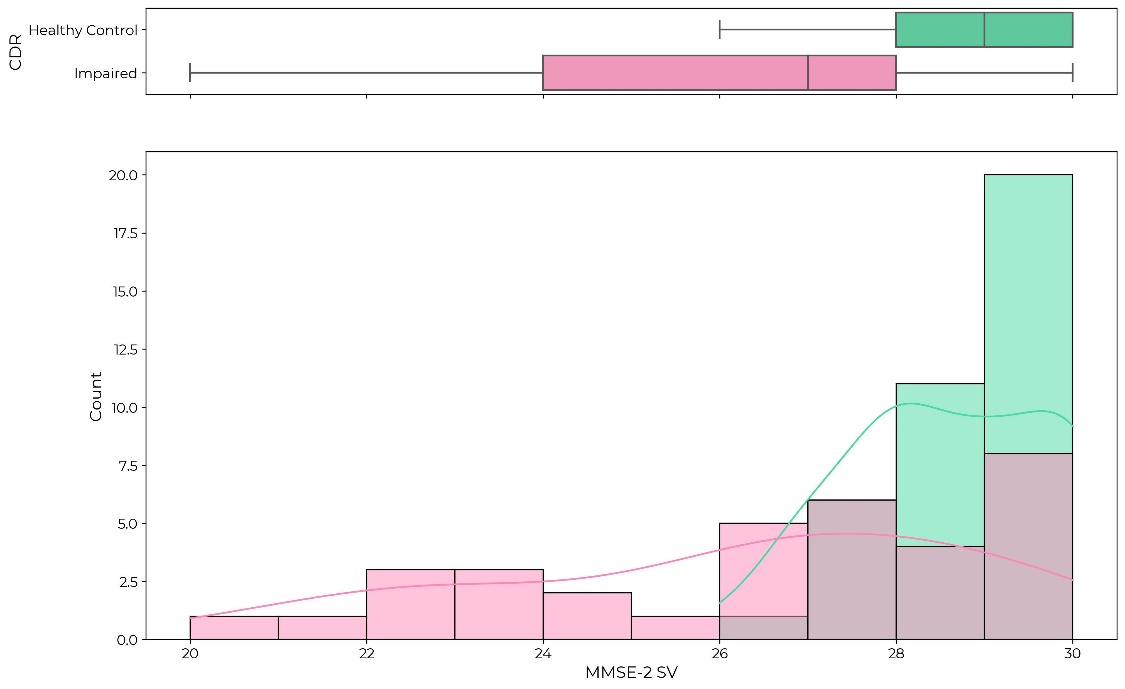


**B**


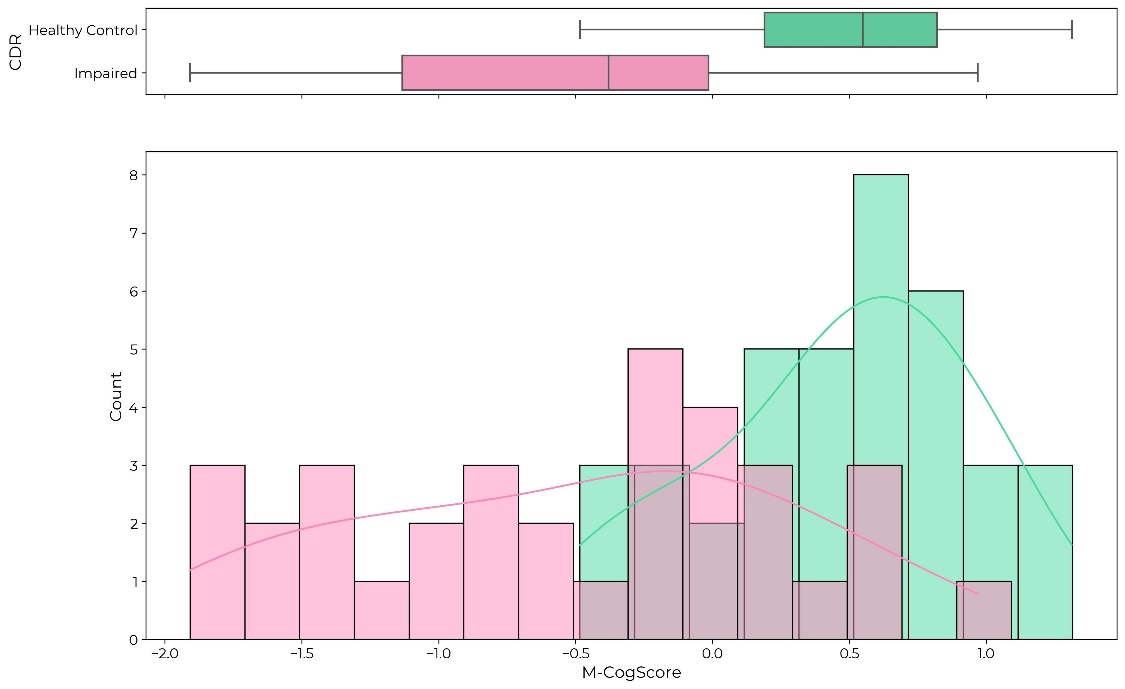


Supplementary Figure 1: distribution of the scores of MMSE-2 (**A**) and M-CogScore (**B**) by CDR label.

## Supplementary Figure 2: distribution of M-CogScore Subscores

**
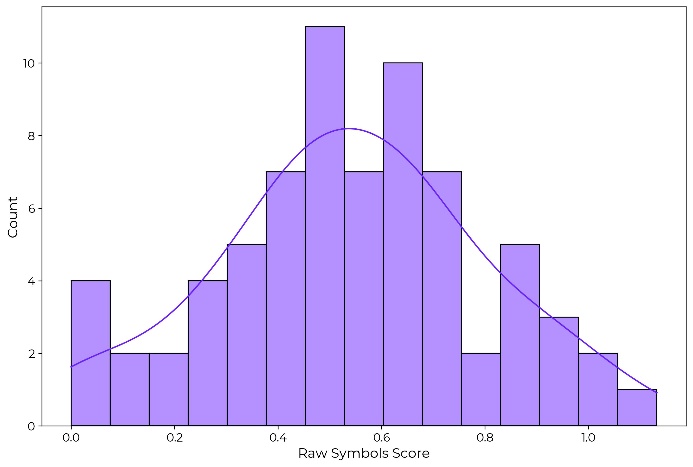

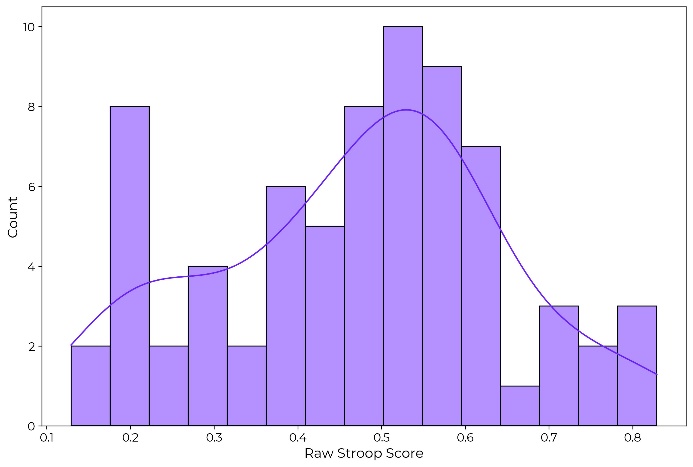

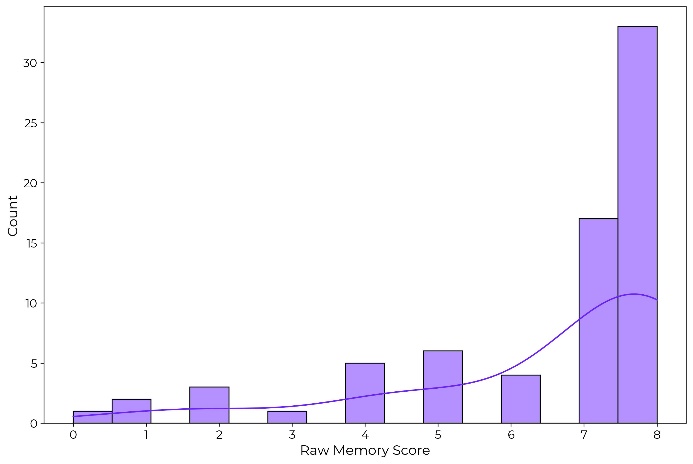
A Memory (delayed recall)** **C Stroop** **E Symbols**

**
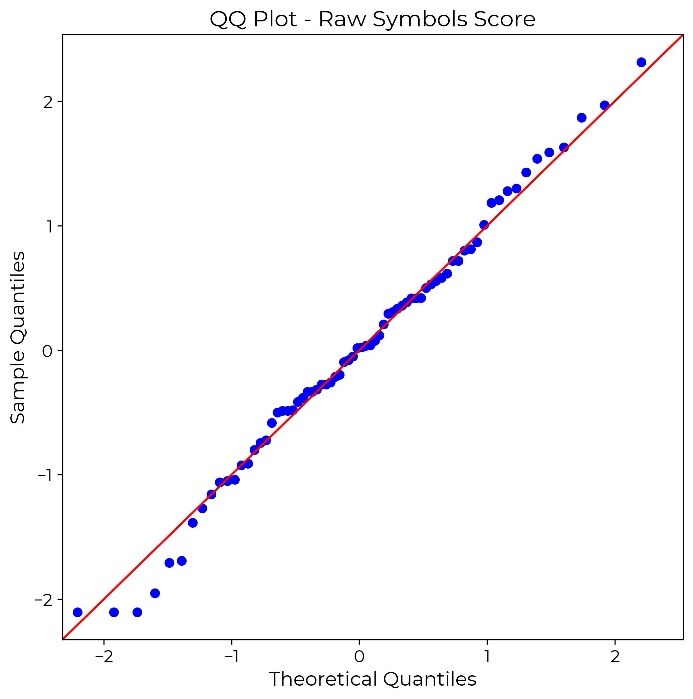

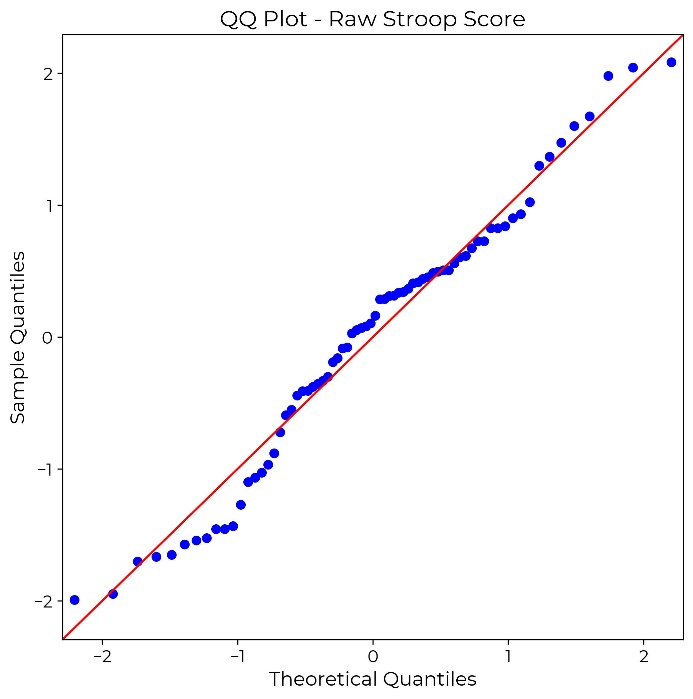

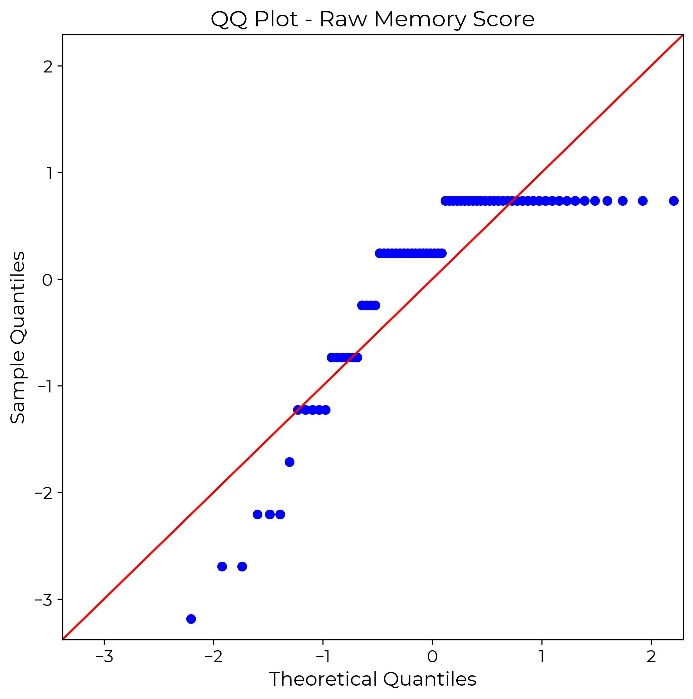
B Memory (delayed recall)** **D Stroop** **F Symbols**

Supplementary Figure 2: distribution of subscores. Memory was not normally distributed (Shapiro-Wilk p<0.05) while Stroop and Symbols were (p=0.10, 0.73, respectively).
